# Phase Variation of LPS and Capsule Is Responsible for Stochastic Biofilm Formation in *Francisella tularensis*

**DOI:** 10.3389/fcimb.2021.808550

**Published:** 2022-01-14

**Authors:** Kevin D. Mlynek, Christopher T. Lopez, David P. Fetterer, Janice A. Williams, Joel A. Bozue

**Affiliations:** ^1^ Bacteriology Division, U.S. Army Medical Research Institute of Infectious Diseases (USAMRIID), Frederick, MD, United States; ^2^ Division of Biostatistics, U.S. Army Medical Research Institute of Infectious Diseases (USAMRIID), Frederick, MD, United States; ^3^ Pathology Division, U.S. Army Medical Research Institute of Infectious Diseases (USAMRIID), Frederick, MD, United States

**Keywords:** biofilm, *Francisella tularensis*, phase variation, viable non-culturable, stochastic, pH effect

## Abstract

Biofilms have been established as an important lifestyle for bacteria in nature as these structured communities often enable survivability and persistence in a multitude of environments. *Francisella tularensis* is a facultative intracellular Gram-negative bacterium found throughout much of the northern hemisphere. However, biofilm formation remains understudied and poorly understood in *F. tularensis* as non-substantial biofilms are typically observed *in vitro* by the clinically relevant subspecies *F. tularensis* subsp. *tularensis* and *F. tularensis* subsp. *holarctica* (Type A and B, respectively). Herein, we report conditions under which robust biofilm development was observed in a stochastic, but reproducible manner in Type A and B isolates. The frequency at which biofilm was observed increased temporally and appeared switch-like as progeny from the initial biofilm quickly formed biofilm in a predictable manner regardless of time or propagation with fresh media. The Type B isolates used for this study were found to more readily switch on biofilm formation than Type A isolates. Additionally, pH was found to function as an environmental checkpoint for biofilm initiation independently of the heritable cellular switch. Multiple colony morphologies were observed in biofilm positive cultures leading to the identification of a particular subset of grey variants that constitutively produce biofilm. Further, we found that constitutive biofilm forming isolates delay the onset of a viable non-culturable state. In this study, we demonstrate that a robust biofilm can be developed by clinically relevant *F. tularensis* isolates, provide a mechanism for biofilm initiation and examine the potential role of biofilm formation.

## Introduction

*Francisella tularensis* is an intracellular Gram-negative bacterium found ubiquitously across the northern hemisphere and is the causative agent of tularemia. Tularemia is most common among small mammals, such as rabbits and voles, and can be transmitted *via* arthropod bites, inhalation or direct contact with an infected organism (Ellis et al., 2002; Sjostedt, 2007). For humans, the glandular and ulceroglandular forms of tularemia are the most prevalent disease manifestations, typically occurring from an arthropod bite. Though less common, pneumonic forms of tularemia acquired from inhalation of aerosolized bacteria pose the most serious threat (Oyston et al., 2004; Hepburn and Simpson, 2008). *F. tularensis* is of particular concern for human health due to its high morbidity, ease of aerosol inoculation and low infectious dose leading to the United States Centers for Disease Control classification as a Tier 1 select agent (Dennis et al., 2001; Keim et al., 2007). Multiple *Francisella tularensis* subspecies have been identified, however, the most consequential to human health are *F. tularensis* subsp. *tularensis* (Type A) and *Francisella tularensis* subsp. *holarctica* (Type B) with the former generally regarded as being more virulent. Within North America, *F. tularensis subsp. tularensis* isolates are typically found in association with more terrestrial environments. In contrast, *F. tularensis subsp. holarctica* tends to be more widely distributed throughout both North America and Eurasia, often in association with aquatic environments (Jellison, 1974; Oyston et al., 2004; Sjostedt, 2007). This divergence in environmental prevalence is also reflected in the associated arthropod vector. Namely, most tularemia cases in the United States are thought to occur from tick bites, while mosquitoes tend to be the drivers of European tularemia cases (Zellner and Huntley, 2019; Tully and Huntley, 2020).

Though tularemia is often associated with rodents and lagomorphs, these populations are not likely to serve as a long-term reservoir of *F. tularensis* as infected individuals either rapidly succumb to disease or clear the infection (Oyston et al., 2004; Telford and Goethert, 2020). It is much more likely that *F. tularensis* persists in the environment outside a mammalian host as this bacterium has been found to maintain viability in an arthropod vector, as well as cold water for extended periods of time (Forsman et al., 2000; Telford and Goethert, 2010; Mani et al., 2015). Further, protozoa have been shown to graze on both Type A and B strains of *F. tularensis* although it is unclear if *F. tularensis* is able to replicate within these host cells (Abd et al., 2003; Thelaus et al., 2009; Buse et al., 2017). However, these environments present unique challenges for the bacterial cell to contend with, such as low nutrient availability, vector immune system and transstadial transmission, as well as environmental fluctuations including pH and temperature.

Over the past few decades, an abundance of work has led to the conclusion that biofilms are a distinct lifestyle that is often integral for survival in array of environments. It is believed that many bacteria found in a natural setting, environmental or pathogenic, are likely in a biofilm state (Stoodley et al., 2002; Hall-Stoodley et al., 2004). These bacterial communities encased within extracellular matrix material (ECM) showcase resilience when faced with barrage of adverse environmental conditions, such as rapid osmolarity changes, nutrient deprivation, or even predation. Most of what we know about biofilm development in Francisella comes from the use of *F. novicida*, a closely related opportunistic pathogen that is routinely used as a BSL-2 lab surrogate. It has been elegantly shown that *F. novicida* is able to form a robust biofilm *in vitro* on a variety of surfaces (Margolis et al., 2010; van Hoek, 2013; Hennebique et al., 2019). While *F. novicida* can be a good model to study Francisella biology, the implied notion is that findings can be applied to *F. tularensis* despite there being stark differences between these two species; most notably virulence and ecology as *F. novicida* rarely causes disease in humans, is thought to be mainly waterborne, and lacks a known mammalian host or arthropod vector (Kingry and Petersen, 2014). Differences also arise in the Francisella Pathogenicity Island (FPI) as *F. tularensis* typically harbors two FPIs while only one island is found in *F. novicida* (Nano et al., 2004; Larsson et al., 2005). Contributing to the differences in pathogenicity, distinct structural modifications in O-antigen (O-ag) are found when comparing *F. tularensis* to *F. novicida* as the core oligosaccharide in *F. tularensis* lacks a glucose residue in the β-glucose branch and the tetra-saccharide repeat is flanked by distinct sugar moieties (Vinogradov et al., 2002; Vinogradov et al., 2004; Gunn and Ernst, 2007). Further, phase variation of the O-ag between blue and grey forms has been described in *F. tularensis*, but this phenomena has yet to be observed in *F. novicida* (Eigelsbach et al., 1951; Soni et al., 2010). Lastly, *F. novicida* has retained the genes necessary to produce cyclic dimeric GMP (cdGMP), a well-known secondary messenger that stimulates biofilm formation (Zogaj et al., 2012). The genes required to synthesize and degrade cdGMP are absent in fully virulent *F. tularensis*, which is thought to confer a selective advantage to the intracellular life-cycle (Zogaj et al., 2012).

A limited amount of studies have examined biofilm formation in both Type A and Type B isolates of *F. tularensis.* These studies found that *F. tularensis* tends to form a biofilm with a sparse cell density over an extended period of time (Margolis et al., 2010; Mahajan et al., 2011; Champion et al., 2019). Recently, Champion et al. used a targeted approach utilizing mutants with deficiencies in O-ag and capsule-like-complex to convincingly show that Type A and B strains are capable of forming a robust biofilm (Champion et al., 2019). Interestingly, it has been well established that *F. tularensis* is able to phase vary components of the lipopolysaccharide (LPS), including the O-ag (Cowley et al., 1996; Hartley et al., 2006; Soni et al., 2010). Indeed, very early studies also understood the importance of phase variation as heterogeneous cultures were found, and phase variants were sorted based on multiple colony morphologies (Eigelsbach et al., 1951; Eigelsbach and Downs, 1961). Variants were sorted into blue (BV) and grey (GV) corresponding to “wild-type LPS” and “altered LPS”, respectively. It was also noted that virulence was severely impacted in GVs using a mouse model challenged intraperitoneally (Eigelsbach et al., 1951). However, a GV identified by Soni et al. retained a similar level of virulence in a mouse model inoculated intranasally (Soni et al., 2010). Given the potential virulence impact, it is unclear how *F. tularensis* may benefit from phase variation.

In this study, we investigate the ability of *F. tularensis* to form biofilm. We show that both Type A and Type B isolates of *F. tularensis* are able to form a robust biofilm that is dependent on a heritable phenotypic switch. We provide evidence that pH may act as a regulator of biofilm development, and biofilm forming cultures remain culturable longer than the parental wild-type. These data presented in this manuscript are the first to describe a potential role for biofilm formation and phase variation of the O-ag in *F. tularensis*.

## Materials and Methods

### Bacterial Strains and Culture Conditions

Bacterial strains used in this study are listed in **Table 1**. *F. tularensis* species were routinely cultured on enriched chocolate agar plates (Remel) at 37°C. For liquid culture, either brain heart infusion broth (BHI) supplemented with 1% IsoVitaleX (Becton-Dickinson), modified Muller-Hinton (MMH) supplemented with 2% IsoVitaleX, or Chamberlain’s Defined Medium (CDM) (Chamberlain, 1965) was used. In some instances, BHI and MMH were pH adjusted as indicated.

**Table 1:** Bacterial strains used in this study.

Strain Designation	Strain characteristics	Source/Reference
LVS	Vaccine strain (Type B)	USAMRIID Repository
FRAN244	Schu S4 (Type A1)	BEI Resources (NR-10492) (Eigelsbach and Downs, 1961)
FRAN249	1958 USAMRIID Schu S4 (Type A1)	BRMR^1^ (Eigelsbach and Downs, 1961).
FRAN250	(Type A1a)	BRMR (Bachert et al., 2021)
FRAN251	(Type A1a)	BRMR (Bachert et al., 2021)
FRAN253	(Type A1a)	BRMR (Bachert et al., 2021)
FRAN254	(Type A1a)	BRMR (Bachert et al., 2021)
FRAN256	Type A2	BRMR (Bachert et al., 2021)
FRAN031	Scherm (Type A1)	BRMR (Downs et al., 1947)
FRAN037	COLL (Type A1)	BRMR (Downs et al., 1947)
FRAN255	Type B	BRMR (Bachert et al., 2021)
FRAN025	VT68 (Type B)	BRMR (Young et al., 1969)
FRAN029	425 (Type B)	BRMR (Bell et al., 1955)
FRAN045	503 (Type B)	BRMR (Olsufiev et al., 1959)
LVS BF+ pop 25	Heterogeneous population of LVS cultured that forms biofilm	Derived by culturing LVS for 7 days in CDM and selected by crystal violet staining.
LVS isolate #9	BV selected from BF+ pop 25,	Derived from LVS during this study.
LVS isolate #10	BV selected from BF+ pop 25,	Derived from LVS during this study.
LVS isolate #11	GV selected from BF+ pop 25,	Derived from LVS during this study.
LVS isolate #12	BV selected from BF+ pop 25	Derived from LVS during this study.
LVS isolate #13	GV selected from BF+ pop 25,	Derived from LVS during this study.
LVS isolate #14	GV selected from BF+ pop 25,	Derived from LVS during this study.
LVS isolate #15	GV selected from BF+ pop 25,	Derived from LVS during this study.
LVS isolate #16	GV selected from BF+ pop 25,	Derived from LVS during this study.
LVS isolate #22	GV selected from LVS based on biofilm formation	Derived from LVS during this study.
LVS isolate #26	GV selected from LVS based on biofilm formation	Derived from LVS during this study.
LVS isolate #27	GV selected from LVS based on biofilm formation	Derived from LVS during this study.
LVS isolate #31	GV selected from LVS based on biofilm formation	Derived from LVS during this study.
LVS isolate #38	GV selected from LVS based on biofilm formation	Derived from LVS during this study.
FRAN244 BF+ 1	GV selected from FRAN244 based on biofilm formation	Derived from FRAN244 during this study.
FRAN255 BF+ 1	GV selected from FRAN255 based on biofilm formation	Derived from FRAN255 during this study.
FRAN255 BF+ 4	GV selected from FRAN255 based on biofilm formation	Derived from FRAN255 during this study.

^1^Biodefense Reference Material Repository.

### Static Biofilm Assay

Francisella strains were cultured for 24 h on chocolate agar and re-suspended to an OD_600_ of 0.3 in phosphate buffered saline (PBS). Bacterial suspensions were then diluted 1 to 10 into fresh CDM (20 μL inoculum into 180 μL of CDM) unless otherwise noted in a CoStar polystyrene 96-well plate seeding the plate with approximately 10^8^ colony forming unit (CFU) per mL. CFU were confirmed by serial dilution and plating throughout this study. Peripheral wells were filled with sterile medium to minimize evaporation over the duration of the experiment and to also provide a sterility control. The plates were then incubated statically at 37°C unless otherwise noted for either 1, 3, 5, 7, 10 or 14 days. The OD_600_ of each well was read prior to staining after which planktonic cells were removed by aspiration, and the remaining biomass was washed 3 times with PBS. The biofilm was fixed for 30 minutes at room temperature in 100% ethanol. Biofilm was visualized by staining for 15 minutes with 0.1% crystal violet stain (Millipore-Sigma) dissolved in H_2_O (w/v) followed 3x PBS washes to remove excess stain. The crystal violet stained biofilm was solubilized in 33% acetic acid (v/v) and the OD_600_ was read using an Infinite M200 Pro (Tecan) microplate reader. When necessary, samples were diluted to ensure OD_600_ were within the linear range of the instrument. Where referred to in text, wells were considered biofilm positive if crystal violet staining was at least 2-fold higher than the sterility control wells with this background subtracted. For instance, if the sterility control wells had an OD_600_ value of 0.25 after staining, an experimental well would need to have a value of at least 0.5 after subtracting the sterility control to be considered biofilm positive.

For progeny experiments and pH crossed experiments, the contents of each well was pipetted vigorously 3x and 20 μL was used to inoculate 180 μL of fresh medium and incubated for 24 h before staining with crystal violet. To determine inoculum CFU, a second aliquot was taken, serially diluted and plated on chocolate agar for enumeration. Where indicated, sterile supernatant from a biofilm positive culture was applied to naïve LVS inoculum. To obtain supernatant, biofilms were grown at 37°C for 7 days in a 24-well plate using a working volume of 1 mL. To harvest, the entire planktonic portion was aspirated into a tube, cells were pelleted by centrifugation and the supernatant was sterile filtered through a 0.22 μm filter.

### Scanning Electron Microscopy

LVS was grown in CDM as described above with the exception that biofilms were grown in a 48-well plate. At the indicated time interval, wells were washed 2x with 0.1 M sodium cacodylate buffer and immediately fixed (2.5% glutaraldehyde, 2% paraformaldehyde in 0.1 M sodium cacodylate buffer, EMSciences) for 1 h at room temperature followed by overnight incubation at 4°C. Samples were then rinsed in buffer and post-fixed with osmium tetroxide for 30 minutes at room temperature. Following post-fixation, samples were washed with 0.1 M sodium cacodylate buffer and dehydrated through a graded ethanol series (30%, 50%, 75%, 85%, and 95%) for 10 minutes each with 3 incubations in 100% ethanol. Samples were further dehydrated using a 1:1 mix of 100% ethanol and hexamethyldisulfide overnight (HMDS; EMSciences). The next day, samples were manually cut from the 48-well plate, mounted on an aluminum stub with carbon and graphite adhesive and sputter coated with platinum. Imaging was carried out using a Zeiss Sigma VP scanning electron microscopy (SEM). To determine the amount of biofilm formed in each well prior to imaging, an additional plate was inoculated by transferring 100 μL of culture into 900 μL of fresh media before fixation. Biofilm in this plate was then visualized by crystal violet staining as described above.

### Detection of LPS and Capsule

Whole cell extracts were prepared by re-suspending cells grown on a chocolate agar plate in PBS to an OD_600_ of 0.5 after which 1 mL of cell suspension was pelleted and washed two times with PBS. After washing, the pellet from LVS was re-suspended in 800 μL PBS with 200 μL NuPage LDS gel loading buffer and boiled for 10 minutes. For other *F. tularensis* strains, pellets were boiled for at least 45 minutes in gel loading buffer and confirmed to be inactivate prior to further analysis. Samples were fractioned on NuPage Novex 4 ± 12% Bis-Tris gels (ThermoFisher). For western analysis, fractionated material were transferred onto a nitrocellulouse membrane using an iBlot Gel Transfer Device. After transfer, the membranes were blocked with 5% skim milk in Tris Buffered Saline + 0.05% Tween 20 overnight. *F*. *tularensis* samples were blotted with mouse monoclonal antibodies, anti-LPS FB-11 (MA1-7388; Invitrogen) or anti-capsule [11B7; (Apicella et al., 2010)], at a dilution of 1:500. Rabbit polyclonal anti- GroEL was used as a loading control at a dilution of 1:2000 (Enzo Life Sciences). Bands were visualized using 3,3’,5,5’-Tetramethylbenzidine Membrane Peroxidase substrate (Kirkegaard & Perry Laboratories Inc).

### Identification of Constitutive Biofilm Forming Isolates

*F. tularensis* was grown for 7 days in a 24-well plate in CDM. The entire contents of the well was disrupted by vigorously pipetting. Next, 900 μL of the cell suspension was mixed with 400 μL of 50% glycerol and stored at -80°C. Biofilm positive wells were identified in the original 24-well plate using crystal violet staining. Biofilm positive freezer stocks were streaked onto chocolate agar and incubated for 2 days to allow identification of individual colonies. Approximately 20-40 well isolated individual colonies were inoculated into 200 μL of CDM and incubated at 37°C. After 3 days, the entire contents of each well were transferred into a fresh replica plate while crystal violet staining was performed on the original. Bacteria from wells identified as biofilm positive were streaked to purity from the replica plate and saved for further analysis.

### Survivorship and Viability Assays

Bacteria were streaked to chocolate agar plates and incubated at 37°C for up to 12 days. At the time of sampling, 3-5 colonies were swabbed from the plate and directly inoculated into liquid medium and swabbed onto a fresh cholate agar plate. At 24 h post inoculation, growth from agar plates was observed, and the OD_600_ was obtained using a spectrophotometer for liquid cultures. Samples were obtained after measuring OD_600_ of the overnight liquid culture and viability was determined using LIVE/DEAD *Bac*Light Bacterial Viability Kit (Life Technologies) following the manufacturer’s instructions. Briefly, 1 mL of culture was pelleted, washed 2x and then stained with 0.17 mM Syto 9 and 1.8 mM propidium iodide for 5 minutes prior to imaging on a Zeiss 700 Confocal Laser Scanning Microscope. Images were collected from at least 2 independent experiments, and representative samples are shown. As a control, select samples were incubated with 70% ethanol for 15 minutes prior to staining.

### Statistics and Reproducibility

**Figure 1:**
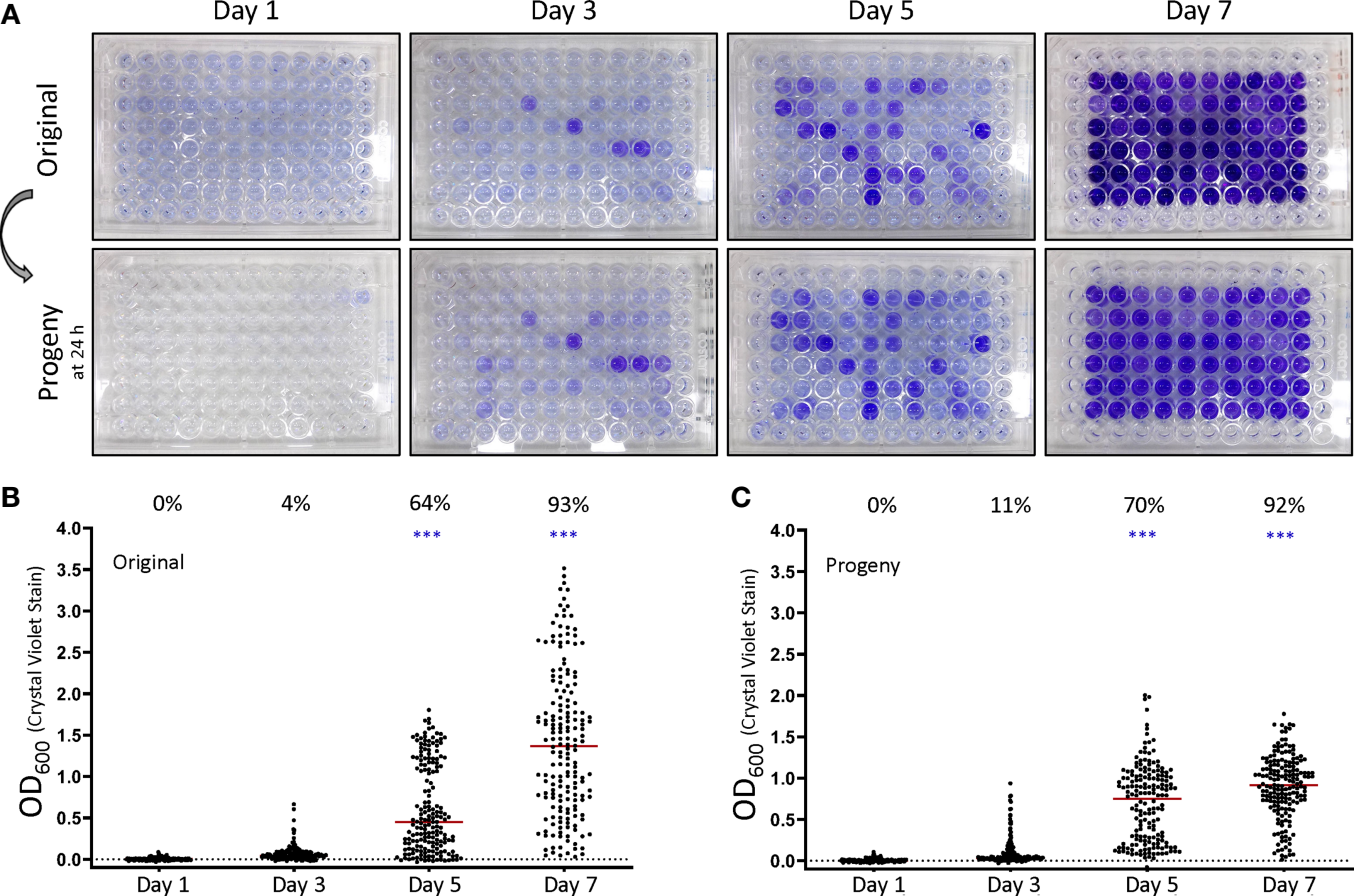
*F. tularensis* subsp. *holarctica* LVS forms biofilm in a stochastic manner that is reproducible in progeny cells. **(A)** Biofilm formation in the original 96 well plates was assessed by crystal violet staining at the indicated time post inoculation. Prior to staining, the original plates were sub-cultured by replicate plating to create progeny plates. Progeny were assayed for biofilm development at 24 h post inoculation. Biofilm was quantitated by determining the OD_600_ values after crystal violet staining for the **(B)** original plates and **(C)** progeny plates. Each point graphed represents an individual well in an experiment. The percent displayed above the graph for each day indicates the percent of biofilm positive wells as described in the materials and methods. The red bar indicates the median value after at least 3 independent experiments. To assess stochastic biofilm formation the log transformed OD_600_ was analyzed by repeated measures linear mixed effects model. ***P < 0.0001.

All experiments implementing statistical analysis described in this manuscript were performed independently at least 3 times. For experiments involving stochastic biofilm formation (**Figures 1**, **3**, **4**, **6**) the log-transformed OD_600_ was analyzed by linear mixed effects model, as described previously (Allkja et al., 2021). Analysis was implemented in the Mixed procedure of SAS version 9.4 (SAS Institute, Inc., Raleigh NC). No multiplicity adjustment was applied, with the exception of **Figure 3**, wherein the adjusted *P*-value, by Dunnett’s method, has been reported. Following testing of normality by Shapiro-Wilk’s test, the comparisons of CFU counts were made by two-way ANOVA with Tukey’s *post-hoc* procedure, as implemented in GraphPad PRISM 8.

## Results

### *F. tularensis* subsp*. holarctica* LVS Forms a Robust Biofilm in a Stochastic But Heritable Manner

Previous studies have shown that *F. tularensis* typically forms a sparse biofilm *in vitro* unless incubated for extended periods (Margolis et al., 2010; Mahajan et al., 2011; Champion et al., 2019). Additionally, mutations have been identified that increase biofilm formation relative to the wild-type (Champion et al., 2019; Siebert et al., 2019; Biot et al., 2020). These data indicate that LVS is capable of developing a dense biofilm and prompted us to assay biofilm formation temporally. To our surprise, during early experiments we found that biofilm formation was highly variable between technical replicates despite the use of a homogenous initial inoculum. To overcome this challenge, the interior wells of an entire 96-plate were inoculated for each experiment. These experiments revealed that LVS can form a strong biofilm in as little as 3 days (~4% of wells), but biofilm forming capacity increased over time with nearly all wells (~93%) forming biofilm by day 7 (**Figure 1A**; top panel, **Figure 1B**). Though biofilm formation was stochastic, a significant number of wells formed biofilm at 5 and 7 days (*P*<0.0001, linear mixed effects model). While most wells formed biofilm after 7 days, a large range in the amount of biofilm formed was still observed. Biofilm formation remained stochastic amongst all experiments in regards to wells forming biofilm within a given plate, but the percentage of biofilm positive wells was surprisingly consistent. Considering all wells in a plate were inoculated from the same inoculum and incubated for the same amount of time, we wondered if the biofilm phenotype would be maintained if sub-cultured into fresh medium. To avoid confounding results from spontaneous biofilm forming wells, progeny were assayed after 1 day of incubation since no biofilm positive wells were observed in the original plates at this time. Progeny plates displayed a similar capacity to form biofilm compared to the original plates and mirrored the staining of the original biofilm plates (**Figure 1A** **;** bottom panel, **Figure 1C**) and displayed similar statistical significance (*P*<0.0001, linear mixed effects model). Of note, the growth as measured by OD_600_ is not indicative of biofilm formation as measured by crystal violet staining as the slope of least squares relating OD_600_ of growth to the OD_600_ of biofilm is near zero **(** **Figure S1A** **)**. Further, no significant differences (*P*>0.45, two-way ANOVA with Tukey’s *post hoc* analysis) were detected in CFU between biofilm negative and biofilm positive samples at days 3, 5 and 7 as biofilm was not observed at time of inoculation or on day 1 of the original plate (**Figure S1B**). Additionally, these data confirm similar CFU were used to inoculate progeny plates at each sampling time in temporal experiments.

To determine if this stochastic biofilm phenotype may occur under lower temperatures, we also assessed biofilm formation at 25°C. These experiments showed that a similar stochastic biofilm phenotype occurs at an ambient temperature, though the time to the initial appearance of biofilm is delayed as biofilm was not observed until day 14 (**Figure S2A**). Further supporting this observation, LVS cultured at 37°C then sub-cultured at 25°C formed biofilm within 24 h (**Figure S2B**). Taken together, these experiments suggest that biofilm could potentially occur at a range of temperatures.

### Biofilm Initiation and Maturation in *F. tularensis* LVS Is Highly Dynamic

**Figure 2:**
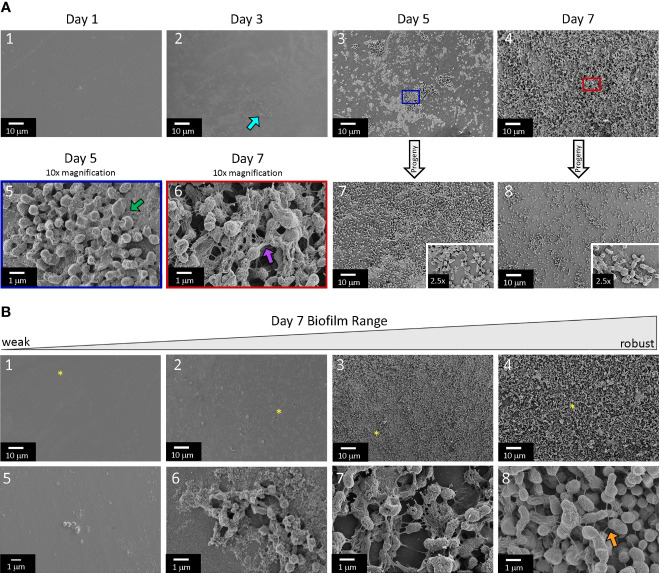
Scanning electron microscopy shows a well-defined extracellular matrix in biofilm positive samples. **(A)** The biofilm of LVS biofilm was sampled at 1, 3, 5 and 7 days and analyzed by SEM. The blue and red rectangle (day 5 and 7, respectively) highlight the enlarged area. Grey arrows labeled progeny indicate the original samples from which cells were sub-cultured to obtain progeny micrographs. Teal arrow indicates surface depositions. Green arrow indicates a smooth, totally encased biofilm. Purple arrow indicates a string-like extracellular matrix. Golden arrow indicates cell to cell projections. **(B)** Representative images of the range of biofilm formation observed in a 7 day culture of LVS. Yellow asterisk (top panel) indicates the area that is enlarged (bottom panel). Images displayed are representative from multiple independent experiments.

Biofilm was visualized for multiple wells at each time point using SEM to assess bacterial colonization and biofilm architecture for LVS (**Figure 2A**). Day 1 samples were devoid of adherent bacteria and had very little deposition of material on the plate surface. Heavy depositions of material or debris that was smaller than bacteria cells were apparent in day 3 samples in nearly all fields of view obtained (teal arrow; moreover, compare Day 3 to Day 1), though minimal colonization of bacteria was detected. By day 5 and 7, some samples still lacked bacterial colonization, though the majority featured a prominent biofilm as detected by SEM. This is in agreement with the crystal violet staining observed in **Figure 1** as not all samples had formed biofilm on Day 5 and 7. To further describe the samples that had prominent biofilm, a broad range of biofilm architecture was evident and, to a lesser extent, differences in biofilm density was even observed within wells (**Figure 2B**). This architecture included cells encased in smooth matrix material (**Figure 2A**, green arrow), and web-like matrix material (**Figure 2A**, purple arrow). Generally, individual bacteria lacking a prominent extracellular matrix (ECM) were observed in areas of heavy surface deposition (**Figure 2B**, panel 5). An ECM became apparent in areas featuring micro-colonies (**Figure 2B**, panel 6), with many of these cell clusters being almost completely encased in a smooth matrix (**Figure 2A** panel 5). In areas of moderate bacterial density, the ECM displayed prominent projections between cells and began to appear more web-like in structure (**Figure 2B**, panel 8). In some instances, cells in direct contact with the plate surface were flattened as the density neared complete confluence (**Figure S3**). It is unclear without further experimentation if these flattened cells were intact or viable. Notably, the densest area of samples showed complete confluence with many layers of cells adhered to the biofilm (**Figure 2B**, panels 3 and 4). Progeny samples generally mirrored what was observed in the original samples, but cells were commonly observed as smooth and encased more often than the in the web-like architecture (**Figure 2A**, panels 7 and 8). Furthermore, we observed a striking increase in the amount of individual cells that were adhered to the plate surface in progeny of biofilm positive samples after 1 day of incubation (compare **Figure 2A**, panel 1 to panel 7). Overall, SEM analysis confirmed the crystal violet staining as broad range of biofilm was observed on day 5 and 7 despite being derived from the same inoculum.

### Fully Virulent Type A and B Strains of *F. tularensis* Are Able to Form Biofilm

**Figure 3:**
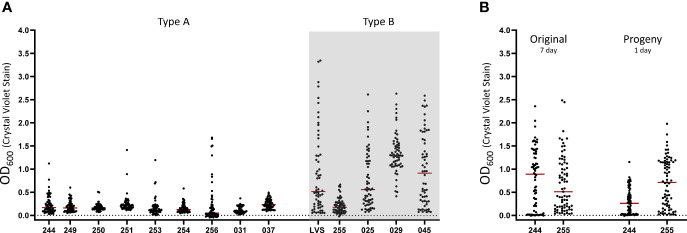
Stochastic biofilm formation is conserved across a diverse panel of fully virulent *F. Tularensis* type A and B isolates. **(A)** The ability of each isolate was assessed using crystal violet staining after 7 days of culturing in CDM in fully virulent Type A (white background) and B (grey background) isolates. LVS was included as a positive control in each assay. **(B)** Replicate plates were inoculated from the original 7 day plates of FRAN244 and FRAN255 (Type A and B isolates, respectively) to assess biofilm formation in progeny cells. Biofilm development was assayed at 24 h post inoculation. Each point graphed represents an individual well in an experiment. The red bar indicates the median value after at least 3 independent experiments.

LVS is an attenuated *F. tularensis* subsp. *holarctica* isolate that is routinely used as a surrogate in BSL-2 laboratories. Though the exact ancestral strain of LVS is unknown, it is thought that attenuation results from the loss of function in a few select genes (Rohmer et al., 2006). We next wanted to test if fully virulent strains of *F. tularensis* shared the stochastic biofilm phenotype observed in LVS. To accomplish this, we screened a diverse panel of fully virulent *F. tularensis* strains that was constructed to aid in the development of future tularemia vaccines (Bachert et al., 2021) as well as three additional Type B strains **(** **Table 1** and **Figure 3A** **).** In these assays, each strain tested (with the exception of FRAN031) had at least one well stain biofilm positive after 7 days (as defined in the *Materials and Methods*). While FRAN031 and FRAN037 appear to form similar amounts of biofilm, FRAN037 met this pre-determined criteria for considering wells as biofilm positive. For the strains we tested, in general, Type A strains switched to biofilm positive at a lower frequency and appear to have a lower mean biofilm forming capacity than Type B strains (*P*<0.01, linear mixed effects model with Dunnett’s method applied). Moving forward, we chose to perform additional experiments using FRAN 244 (Type A; Schu S4 available from BEI) given the ubiquity of the use of this strain in biodefense research and FRAN255 (Type B) given the extensive characterization of it in our lab (Bachert et al., 2021). Using these two strains, we next assayed the ability of progeny cells to form biofilm upon back-dilution. Consistent with LVS studies, both FRAN 244 and FRAN 255 formed a robust biofilm in 24 h that mirrored the original plate (**Figures 3B** and **S4**).

### pH Acts as an Environmental Checkpoint for Biofilm Formation

**Figure 4:**
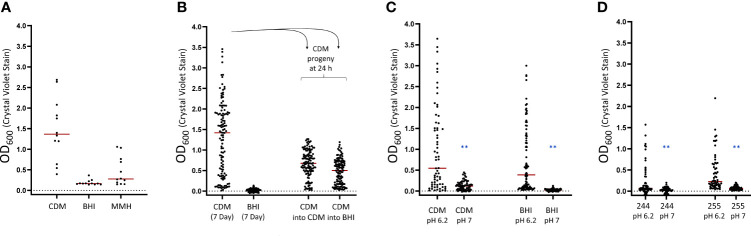
pH of the culture medium impacts the ability of *F. tularensis* to form biofilm. **(A)** LVS biofilm formation was assessed in Chamberlain’s defined medium (CDM), brain-heart infusion broth (BHI) supplemented with 1% IsoVitaleX and modified Mueller-Hinton broth (MMH) supplemented with 2% IsoVitaleX. Biofilms were assayed at 10 days post-inoculation. **(B)** LVS grown in either CDM or BHI for 7 days was sub-cultured *via* replica plating into CDM or BHI. **(C)** LVS biofilm formation was assessed after culturing for 7 days in CDM or BHI pH adjusted to 6.2 or 7. **(D)** The effect of culture medium pH was assessed in fully virulent Type A and B isolates (FRAN 244 and FRAN255, respectively). The red bar indicates the median value after at least 3 independent experiments. **P < 0.01.

Initial screens to determine the conditions that foster biofilm development in LVS included using BHI (Shanson and Singh, 1981), MMH as well as CDM (Chamberlain, 1965). The stochastic formation of biofilm was observed when LVS was cultured in either CDM or MMH (92% and 42% positive, respectively) but not in BHI when cultured for 10 days (**Figure 4A**). BHI has been shown to induce protein expression profiles in *F. tularensis* similar to those of nutrient depleted bacteria (Holland et al., 2017) and mirrors the phenotype of macrophage grown *F. tularensis* (Hazlett et al., 2008). With this in mind, it was unexpected that biofilm was not observed in BHI grown cells as one may expect natural environmental conditions to be oligotrophic. Given that biofilm may provide an advantage in the environment, we performed additional experiments to determine if biofilm can be formed in BHI. Curiously, when LVS was grown in CDM and cells were sub-cultured into BHI, the progeny formed biofilm to levels similar to that observed in progeny experiments performed using CDM within 24 h (**Figure 4B**). This result suggested to us that biofilm initiation was blocked in BHI, but biofilm development can continue if a critical checkpoint is passed. Given that BHI is a complex medium, it is difficult to control the exact chemical composition. However, when comparing the pH of the three media tested, we noticed that the pH of CDM and MMH was near 6 while BHI was slightly above 7. To test the hypothesis that pH may act as a checkpoint for biofilm development, LVS was cultured in BHI or CDM adjusted to 6.2 and 7, respectively. The results of this experiment revealed that stochastic biofilm phenotype was pH dependent as biofilm was observed at pH 6.2, but absent in pH 7 for both CDM and BHI (**Figure 4C**; *P*<0.01, linear mixed effects model). Further, this result was able to be recapitulated using fully virulent Type A and B strains cultured in CDM pH 6.2 or 7 (**Figure 4D**; *P*<0.01, linear mixed effects model). Taken together, these findings are consistent with the notion that pH may function as an environmental checkpoint for biofilm formation.

**Figure 5:**
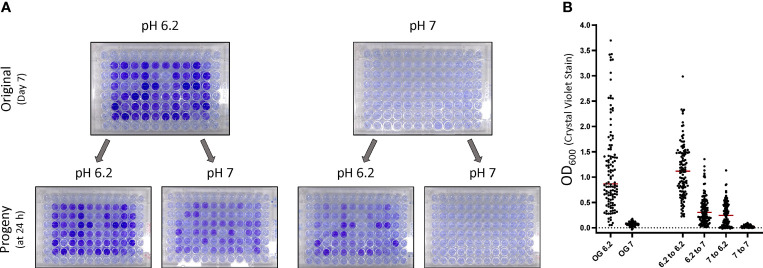
pH acts as an environmental checkpoint for initiation of biofilm matrix assembly. LVS biofilms were grown in CDM adjusted to the indicated pH. After 7 days, bacteria were sub-cultured into either CDM at pH 6.2 or 7 and the progeny biofilms were stained at 24 h. **(A)** Representative images of biofilm plates crossed into CDM at each pH. **(B)** Graphed data after 3 independent experiments. Red bars indicates the median. OG indicates the original plates from which progeny plates were inoculated.

While these experiments demonstrated that a higher pH can inhibit biofilm formation, it was unclear if the pH threshold completely impeded biofilm matrix assembly or prevented biofilm initiation by the bacteria. To answer this question, LVS was cultured for 7 d in CDM at either pH 6.2 or 7 and sub-cultured *via* replica plating into fresh culture medium at each pH. The progeny wells were then assayed for biofilm development at 24 h post inoculation. This experiment revealed that LVS cultured at pH 7 was able to form biofilm if the pH was decreased to 6.2 upon sub-culturing, indicating that the bacteria were likely primed for biofilm formation, but an external factor was likely preventing the development (**Figure 5**). Notably, when biofilm forming wells were crossed from pH 6.2 to pH 7, a decrease in the amount of biofilm was formed, further supporting the importance of the pH for matrix assembly.

### An Inherent Cell Trait Is Responsible for Biofilm Forming Capacity in LVS

**Figure 6:**
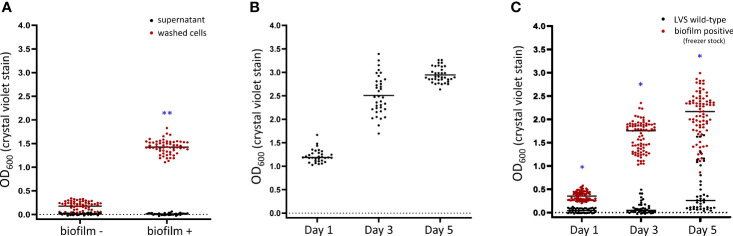
An inherent cell associated trait is responsible for biofilm formation in *F. tularensis*. **(A)** LVS was cultured for 7 days in CDM after which the contents of a biofilm negative and biofilm positive wells were separated into sterilized supernatant or repeatedly washed cells. The sterilized supernatant was applied to naïve cells and the ability to form biofilm was compared to washed cells only using crystal violet staining. The biofilm capacity of the **(B)** washed cells or **(C)** frozen biofilm positive stocks was assayed at 1, 3 and 5 days post-inoculation. Black bars indicates the median. *P < 0.05; **P < 0.01.

Given the stochastic nature and time for initial biofilm development, we wondered if a secreted factor, such as a quorum signal or metabolite, was required and/or a critical threshold surpassed before biofilm development was initiated. To test this hypothesis, we harvested the entire contents of biofilm negative and robust biofilm forming wells of LVS at 7 days, and each sample was separated into either filter sterilized supernatant or washed cell mass. These components were inoculated into fresh CDM medium (washed cells) or spiked into CDM inoculated with naïve LVS (supernatant) and incubated for 24 h before analysis. LVS samples spiked with 10% conditioned supernatant failed to induce biofilm regardless of the origin of the supernatant (**Figure 6A**, black circles). It is important to note that the final cell density at the time of sampling as measured by OD_600_ suggests that growth was not impaired by this treatment. Additional concentrations of supernatant (25% and 50%) were also tested, and no biofilm induction was observed (data not shown). In stark contrast, biofilm positive washed cells quickly formed a robust biofilm in the 24 h timespan (**Figure 6A**, red circles; *P*<0.05, linear mixed effects model). These results suggest that nutrient replete conditions and cell division does not reset the biofilm capacity of positive samples.

The density of the culture (OD_600_) obtained at 24 h indicated that the cells were likely still in the exponential phase of growth as the average value was approximately half of what is observed at day 3 (**Figure S5**). Additional experiments were performed to measure biofilm formation of the progeny 3 and 5 days post inoculation to determine if the biofilm continued to increase into stationary phase. These experiments revealed that biofilm continued to increase at days 3 and 5 (**Figure 6B**). Taken together, these results support that biofilm capacity is due to a cell associated trait. We next wondered whether the initiation of biofilm formation was stored at the DNA or RNA level in these samples. To test this, glycerol stocks of biofilm positive cells were made and stored at -80°C. A biofilm assay was then repeated using these stored isolates as described in the Materials & Methods, and biofilm formation was measured at 1, 3 and 5 days. To our surprise, biofilm positive isolates retained an increased biofilm capacity and formed biofilm similar to what was observed before freezing (**Figure 6C**).

### Phase Variation Is Readily Observed in Cultures Capable of Forming Biofilm

When streaking LVS biofilm forming cultures from long term freezer stocks onto a chocolate agar plate, it became clear that multiple colony morphologies were present. Colonies displayed variation in the color ranging from slightly opaque to brilliant white while there was also differences in colony size after 2 days of growth (**Figure S6**, red arrows compared to black). Studies in the 1950’s with *F. tularensis* showed that this bacterium can phase vary from a “blue” to “grey” form based on colony appearance under oblique lighting (Eigelsbach et al., 1951). It was later determined that this blue/grey phase variation is due to structural changes in the LPS, often in the O-ag (Cowley et al., 1996; Hartley et al., 2006; Soni et al., 2010). Given that multiple colony morphologies were observed in the freezer stock of our LVS biofilm positive population, we suspected that phase variation may occur giving rise to the stochastic biofilm phenotype observed.

**Figure 7:**
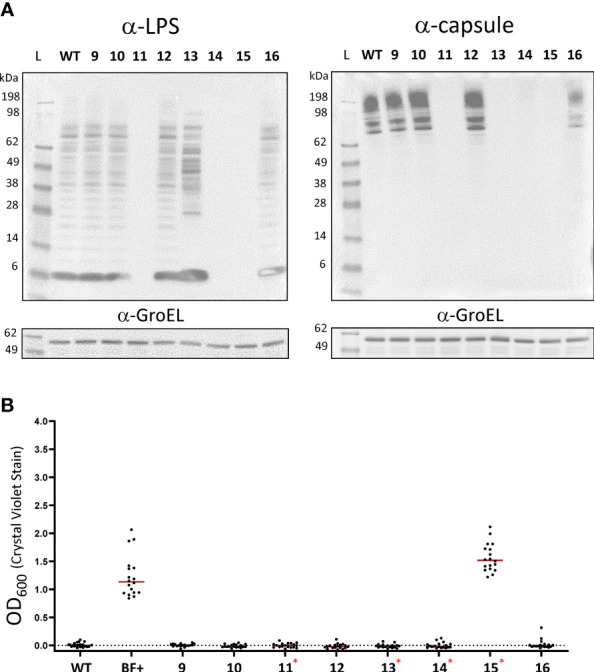
Blue-grey phase variation is readily observed in biofilm forming cultures. **(A)** Western blot analysis of LVS strains was performed on 8 representative colony variants that arose after streaking biofilm positive freezer stocks on chocolate agar. Pellets from purified isolates were lysed and equal concentrations of extracts were separated on SDS-PAGE gels blotted with either α-LPS (left) or α-capsule (right). In each case, α-GroEL was used as a loading control. WT corresponds to the wild-type LVS strain. Numbers correspond to the respective variant isolates. **(B)** A crystal violet biofilm assay was used to determine the biofilm forming capacity of each of the purified LVS isolates. Red asterisks indicate grey variants as identified by western blotting. Red lines indicate the median.

To determine if the biofilm phenotype could be attributed to a particular colony morphology present in the biofilm forming culture, representative colonies with a range of morphologies were streaked to purity. Whole cell extracts were prepared from each purified isolate of the biofilm forming freezer stock, and western analysis was performed using α-LPS or α-capsule monoclonal antibodies. To our surprise, 4 out of 8 purified isolates had an altered LPS and/or capsule structure as demonstrated by the banding pattern with the respective antibody directed against these structures. Therefore, these data indicate that these isolates are grey varied (**Figure 7A**). Next, these purified isolates were assayed for biofilm development at 3 days as it was rarely observed during this time in previous experiments for wild-type cultures. This experiment revealed that a small opaque purified isolate in particular, isolate 15, formed nearly identical levels of biofilm as the biofilm positive stock culture (**Figure 7B**). Further, isolate 15 was among the grey varied isolates as it did not react with either the antibody to LPS or capsule. Taken together, these results indicate that grey variation is readily observed in biofilm forming cultures and also demonstrate that not all grey variants are capable of forming biofilm.

**Figure 8:**
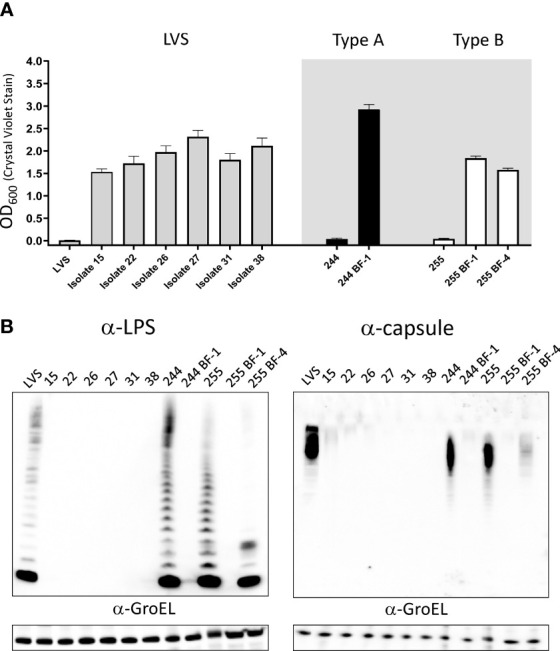
Grey variants are responsible for the production of biofilm in *F. tularensis*. **(A)** Biofilm formation of purified constitutive biofilm forming isolates assayed by crystal violet staining after 3 days of growth in CDM. Grey shaded area highlights fully virulent Type A and B *F. tularensis* isolates and the isogenic biofilm former. **(B)** Western blot analysis was performed on the purified biofilm forming isolates to assess LPS and capsule. Bacteria were suspended to an equal OD_600_. Cell pellets were lysed and were separated on SDS-PAGE gels blotted with either α-LPS (left) or α-capsule (right). Equal amounts of whole cell extracts were loaded, with the exception of 244 and 255 as these isolates were diluted 1:4 and 1:2, respectively. In each case, α-GroEL was used as a loading control.

We next wondered if all biofilm forming isolates were grey variants. To bolster the sample size of biofilm forming isolates, additional biofilm positive populations of LVS, FRAN 244 and FRAN 255 (fully virulent Type A and B, respectively) were identified and saved. To identify purified biofilm positive colonies, approximately 20 well isolated colonies from each of the population stocks were inoculated into CDM and incubated for 3 days after which biofilm was assayed using crystal violet. Biofilm positive wells were then plated and streaked to purity after which the ability to form biofilm was reconfirmed (**Figure S7**). Each of these biofilm forming isolates featured the small opaque colony morphology regardless of strain background (representative image in **Figure S7D**). All isolates formed a robust biofilm in 3 days when the ability to form biofilm was tested (**Figure 8A**). Western analysis using α-LPS and α-capsule monoclonal antibodies demonstrated that each of these biofilm forming isolates were also grey varied when compared to the wild-type controls (FRAN244 and FRAN255, labeled 244 and 255 respectively) (**Figure 8B**). Of note, FRAN255 isolate 4 maintained some level of staining for both α-LPS (FB11) and α-capsule (11B7) antibodies but was clearly altered as compared to the parent profile. Taken together, these data indicate that all biofilm formers identified in this study are grey variants, but not all grey variants are biofilm formers.

### Biofilm-Positive Cultures Delay the Onset of a Viable Non-Culturable State

**Figure 9:**
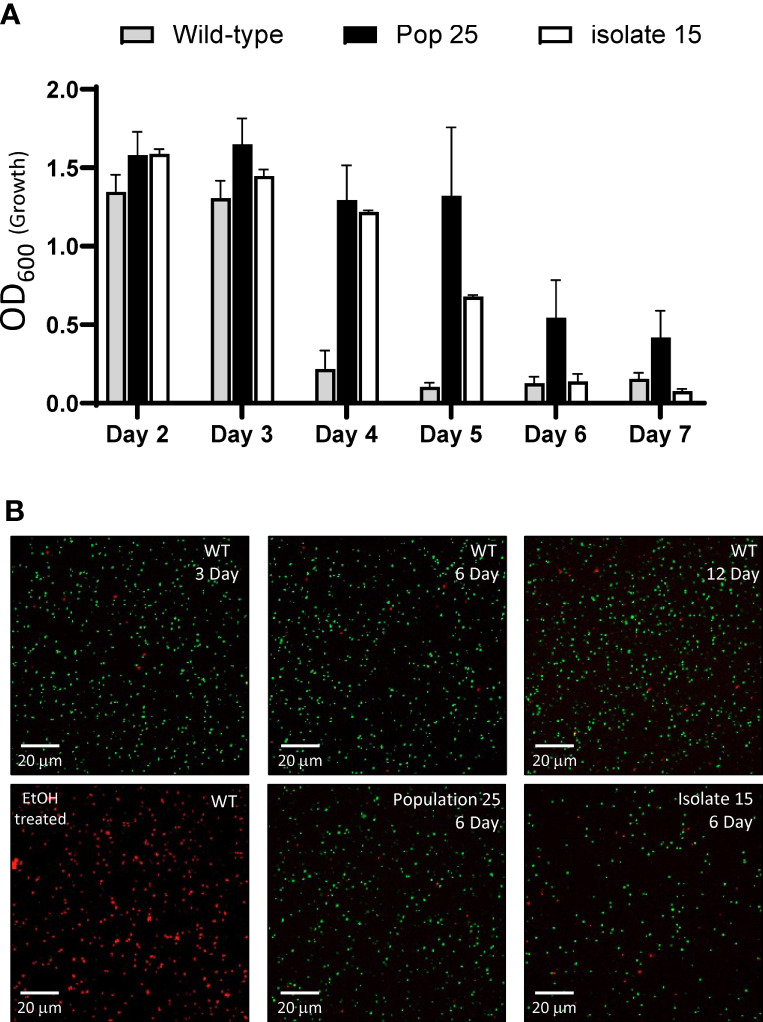
Biofilm forming isolates delay the onset of a viable, but non-culturable state. **(A)** *F. tularensis* LVS (grey), a biofilm forming population (black) and a purified constitutive biofilm forming isolate (white) derived from wild-type LVS were streaked onto chocolate agar and incubated at 37°C. At the time indicated, CDM was inoculated and incubated shaking at 37°C for 24 h. The OD_600_ was obtained as a measurement of bacterial growth. Samples were diluted as required to maintain the linear range of measurement. Graphed data show the average of at least 3 independent experiments with the standard error of the mean. **(B)** An aliquot of the bacteria present from the 24h broth culture was assayed for viability using Live/DEAD stain followed by fluorescent microscopy. In these representative images, bacteria with an intact membrane (live) stain green while those with an impaired membrane (dead) are indicated by red staining. LVS exposed to 70% EtOH for 15 minutes was stained and imaged alongside samples to serve as a control.

Biofilm is thought to be important for the persistence of bacteria in host tissue, arthropod vectors and environmental reservoirs for many bacteria (Hall-Stoodley et al., 2004; Hinnebusch and Erickson, 2008; Lutz et al., 2013). In *F. novicida* and *F. philomiragia*, biofilms have been shown to extend survival in water when compared to planktonically grown cells (Siebert et al., 2020). In our hands, we noticed that when streaked on an agar plate, LVS requires 2 days for individual colonies to become visible, but after 3+ days of incubation at 37°C, these colonies become difficult to culture by swabbing onto a subsequent plate or inoculation into broth. With this in mind, we hypothesized that biofilm formation may increase the longevity and recoverability of *F. tularensis* cultures. To test this hypothesis, the recoverability of LVS wild-type was compared to biofilm forming strains (a biofilm forming population, #25, and a biofilm positive isolate, #15). All strains were streaked to choclate agar plates and incubated at 37°C. Each day, a sample was obtained by inoculating fresh CDM broth and incubating at 37°C with shaking. Growth was assayed by obtaining the OD_600_ for each sample at 24 h post-inoculation. This experiment revealed that wild-type cells failed to grow 3 days post streaking while a mixed population (Pop 25) was able to be grown reliably up to 5 days post streaking (**Figure 9A**). In some experiments, growth was still observed in Pop 25 on day 6 and 7 post streaking. A biofilm forming isolate (#15) purified from Pop 25 also remained culturable 5 days post streaking. However, no growth was observed past 5 days (**Figure 9A**). In parallel experiments, applicator swabs used to inoculate CDM shake cultures were swabbed on fresh chocolate agar plates to assay static growth. The results of these experiments were in agreement with CDM broth experiments (data not shown).

Given that a viable but non-culturable state has been previously identified in *F. tularensis*, the viability of bacteria present in the cultures was assayed *via* microscopy using LIVE/DEAD staining (Forsman et al., 2000; Siebert et al., 2020). Consistent with a viable non-culturable state, the majority of the bacteria in inoculated broth cultures for all of the strains tested were alive as indicated by absence of propidium iodide staining (**Figure 9B**). LVS wild-type as well as population 25 and isolate #15 was found to be viable at day 3 (growth observed) and 6 (no growth observed). Additionally, LVS wild-type was sampled at 12 days post streaking and the vast of majority of the bacteria were found to be viable as indicated by LIVE/DEAD staining. These experiments demonstrate that biofilm forming isolates of *F. tularensis* can delay the onset of a viable non-culturable state in *F. tularensis.*

## Discussion

Biofilm formation is thought to be the predominant lifestyle of bacteria found in the environment as the ECM affords the bacteria protection from hostile conditions and the altered metabolic activity associated with biofilm provides a buffer to nutrient stress. In particular, the ability of Francisella species to form biofilm has been questioned, namely because biofilms formed by pathogenic species have been described as being typically sparsely populated, erratic or weak when performed in a short time-frame *in vitro* (Margolis et al., 2010; Mahajan et al., 2011; Champion et al., 2019). However as we show from this study, we were able to observe robust biofilm formation, but in a stochastic manner from a diverse set of *F. tularensis* strains to include both Type A and B strains. Other key takeaways from our study are demonstrating the ability of a *F. tularensis* culture to form biofilm was dependent on both pH and conversion to grey variants. Finally, we identified a subset of LVS *F. tularensis* grey variants as being able to constitutively form biofilm and also delay in the ability to revert to a viable non-cultural state when compared to the parent strain.

The ability of the closely related Francisella surrogate strain *F. novicida*, which is a strict aquatic organism and not normally pathogenic for humans, to form biofilm is well established, and biofilm most likely provides this organism the ability to persist in the environment (Durham-Colleran et al., 2010; Margolis et al., 2010; Champion et al., 2019). Also, the *F. novicida* genome has a gene cluster that encodes for proteins possessing diguanylate cyclase (DGC) and phosphodiesterase (PDE) domains involved in the synthesis and degradation of the secondary messenger cyclic di-GMP (cdGMP) (Zogaj et al., 2012). cdGMP is a secondary messenger associated with controlling biofilm formation, along with other bacterial cellular processes (Tamayo et al., 2007; Boyd and O’Toole, 2012).

Type A and Type B *F. tularensis* isolates also have the ability to also reside in the environment, but in contrast to *F. novicida*, these Francisella species are highly pathogenic for higher level mammals. Furthermore, these pathogenic species of Francisella, as opposed to *F. novicida* described above, are missing the genes encoding the proteins involved in regulating the cdGMP (Zogaj et al., 2012). Therefore, the ability and exact role of biofilm in survival or virulence for these Francisella species has been questioned. If biofilm plays a role in persistence and transmission of Francisella remains unknown, largely because most biofilm studies have been completed with *F. novicida* (Tully and Huntley, 2020).

In this study, we demonstrate that a robust biofilm can be formed quickly by Type A and Type B *F. tularensis* isolates despite the absence of the cdGMP signaling system. This finding alters the perception that these subspecies typically only form a low density, weak biofilm and has implications for future studies on the role of biofilm in virulent Francisella subspecies. Furthermore, this work also lends support to the notion that pathogenic Francisella species do not rely on a continuous infection cycle of vertebrates to serve as an environmental reservoir (Telford and Goethert, 2020). Most bacteria in the environment are thought to be found in biofilm communities rather than free-living planktonically (Costerton et al., 1978; Hall-Stoodley et al., 2004). Often with biofilm formation the bacterium inhabitants alter their metabolism, usually in favor of a quiescent state (Stoodley et al., 2002). *F. novicida* has already been shown to readily form a robust biofilm on a variety of surfaces, including chitin, and it has been suggested that N-acetyl-D-glucosamine (GlcNAc), the end product of chitin hydrolysis, can serve as a carbon source in the absence of glucose (Margolis et al., 2010). Notably, chitin is present within the exoskeleton of arthropods, such as ticks and mosquitoes, which are known vectors for transmission of tularemia. Thus, the question arises if *F. tularensis* present within arthropod vectors are in a biofilm?

Additionally, we show in this study that while both Type A and B isolates stochastically formed biofilm, Type B strains appeared to more readily produce this product. Though, we only tested 5 isolates of Type B strains, perhaps this increased biofilm formation is due to the more aquatic based ecological niches typical for this subtype of *F. tularensis*. Distinct differences exist in the vector ecology between *F. tularensis* (Type A) and *F. holarctica*, (Type B), particularly in the United States. While Type A and B strains are found distributed across North America, Type A isolates are closely associated with lagomorphs and ticks often in more arid terrestrial settings. In contrast, Type B isolates are associated with deer flies, mosquitoes and rodents in aquatic conditions (Staples et al., 2006; Keim et al., 2007; Kugeler et al., 2009). Mosquito larvae have also been found to readily graze on LVS biofilms in water, perhaps fostering environmental persistence as the bacteria were found to escape the midgut and colonize this host (Mahajan et al., 2011). However, it has been suggested that mosquitoes and deer flies are not long term reservoirs of *F. tularensis* (Sjostedt, 2007; Maurin and Gyuranecz, 2016). Microorganisms, such as protozoa, including free-living amoeba, have been shown to graze on both Type A and B strains of *F. tularensis* resulting in enhanced colonization of these organisms in water, though it is unclear if replication of *F. tularensis* occurs and/or is host specific (Abd et al., 2003; Thelaus et al., 2009; Buse et al., 2017).

A recent study by Golovliov et al. found that Type A and B isolates of *F. tularensis* failed to produce biofilm in 0.9% saline (Golovliov et al., 2021). However, it is difficult to compare this study to ours given that biofilm formation is a metabolically active process employing the synthesis of macromolecules to build the ECM (Mann and Wozniak, 2012; Hobley et al., 2015). Bacteria present in a natural aquatic setting would have more nutrients available than saline. Furthermore, bacteria are more likely to be present within the sediment in an aquatic environment which would also contain some level of nutrients. Certainly, further studies are needed to determine if *F. tularensis* forms biofilm in a true environmental setting and if biofilm aids or hampers the colonization and/ or predation of protozoans. Biofilm can provide protection form predation by effectively “bulking up” colony morphology, however, biofilm also concentrates bacteria allowing for a higher inoculum when contact occurs with other organisms (Costerton et al., 1999; Darby et al., 2002; Matz and Kjelleberg, 2005).

Curiously, another mechanism employed by bacteria to control predation pressure is the variation of the O-ag (Wildschutte et al., 2004). LVS previously was shown to establish a biofilm of considerable biomass after 15 days incubation in Mueller-Hinton broth, though it was noted that significant variations were observed across the biofilm (Mahajan et al., 2011). In our studies, the well to well variation observed was a unique phenotype that is likely due to phase variation and, as our data suggests, a sub-population of GVs are driving biofilm formation. Phase variation was described early during the characterization of Francisella virulence (Eigelsbach et al., 1951), and it was later found that the O-ag was altered in GVs (Cowley et al., 1996; Hartley et al., 2006). GVs have also been reported to revert back to the BVs, though the underlying genetic mechanism has not been elicited (Eigelsbach et al., 1951; Soni et al., 2010) Given that the Francisella LPS structure is unique and is critical for pathogenesis (Raynaud et al., 2007; Kim et al., 2012; Jones et al., 2014; Rasmussen et al., 2014), it is difficult to understand why phase variation readily occurs in Type A and B isolates of *F. tularensis*. Indeed, numerous studies have shown that GVs are attenuated to at least some degree (Eigelsbach and Downs, 1961; Cowley et al., 1996; Hartley et al., 2006; Soni et al., 2010). Our data provides a possible explanation as *F. tularensis* may undergo phase variation, despite the cost of virulence, to enter a biofilm lifecycle. Heterogeneity in the O-ag produced has been observed in numerous species, including the intracellular pathogens, such as *Brucella abortus*, *Legionella pneumophila*, and *Burkholderia pseudomallei* (Freer et al., 1995; Luneberg et al., 1998; Tuanyok et al., 2012). Though the exact mechanism responsible for phase variation of the O-ag promoting biofilm in Francisella is yet to be discovered, the O-ag is largely responsible for cell surface attributes, such as hydrophobicity and surface charge, and has been implicated in biofilm formation in Gram-negatives. For instance, *Pseudomonas aeruginosa* produces two distinct types of O-ag (common polysaccharide antigen [CPA] and O-specific antigen [OSA]) when grown planktonically, but as the transition to a robust biofilm occurs, the length of CPA is decreased or lost, ultimately promoting cell to cell adhesion and surface attachment (Lam et al., 1989; Lindhout et al., 2009). Furthermore, it was found that the decrease of CPA occurs in a dependent cdGMP manner (McCarthy et al., 2017). Additional studies have observed an increase in cell surface hydrophobicity and the secretion of outer membrane vesicles (OMVs) for *P. aeruginosa* (Baumgarten et al., 2012).

Perhaps a link between OMVs and biofilm formation exists for *F. tularensis*. In LVS, increased OMV secretion and biofilm formation was observed in a *fupA* mutant (Siebert et al., 2019). Additional experiments performed by Siebert and colleagues demonstrated that the addition of OMVs increased biofilm formation in a dose dependent manner (Siebert et al., 2019). Interestingly, a link between GVs and OMVs exist as a GV with extended O-ag was found to form more membrane vesicles compared to wild-type LVS (Soni et al., 2010). Further supporting this link, O-ag mutants were found to make smaller vesicles (Champion et al., 2018).

In this manuscript, we also demonstrate that pH can act as an environmental checkpoint for biofilm formation. The result that *F. tularensis* cultured at pH 7 does not form biofilm, but upon sub-culture to a slightly acidic pH a robust biofilm is formed quickly suggests that matrix assembly is impeded and phase variation is unaffected. While at this time we are unable to rule out that pH effects a critical enzyme or signal molecule for biofilm formation, it is more likely that surface adherence is affected. In *F. novicida*, chitinase was found to affect the biophysical properties to control adhesion and biofilm production by increasing the cell surface charge to foster interactions with negatively charged surfaces (Chung et al., 2014). A study by Champion *et al.*, convincingly demonstrated that a double mutant lacking O-ag and the capsule like complex adhered significantly better than wild-type and formed a robust biofilm (Champion et al., 2019). The results of our western analysis shows that all of the naturally occurring variants identified in our study have an altered O-ag and capsule, consistent with the results presented by Champion et. al. In their study, Champion et al. found that biofilm formation was dependent upon growth medium and that BHI grown bacteria produced less biofilm (Champion et al., 2019). Here, we show that biofilm can be quite robust in BHI if the pH is decreased, suggesting that nutritional differences alone do not account for observed lack of biofilm, but rather the chemical properties of the medium can impact biofilm formation. Given that it is unclear how phase variation that results in biofilm specifically alters the O-ag, further studies are needed to resolve the structural differences in these variants to understand the mechanistic details. However, both the growth environment and nutrient availability likely play a decisive role in cell fate as Francisella enters a VBNC.

Free-living planktonic *F. tularensis* has been shown to quickly lose the ability to be detected by culturing when grown in fresh water (Berrada and Telford, 2011). *F. tularensis* has been shown to remain metabolically active in water despite being undetectable by culturing on agar plates (Forsman et al., 2000; Gilbert and Rose, 2012). A recent study by Siebert et al., has shown that biofilms of *F. novicida* and *F. philomiragia* allow the bacteria to survive longer than those grown planktonically (Siebert et al., 2020). In agreement with this study, we found that LVS populations that contain GVs that constitutively produce biofilm, the onset of the VBNC state is delayed. While further studies are needed, this result suggests that the metabolic state of biofilm forming isolates is different from that of wild-type cells, providing metabolic heterogeneity to the population. One possibility is that heterogeneity of BV and GV is likely important particularly during overwintering when detection of infections in mammals are low (Jellison, 1974; Mani et al., 2016). Indeed, *F. tularensis* has been found to modify the acyl chains of lipid A in response to temperature fluctuation (Phillips et al., 2004; Li et al., 2012). Phenotypic heterogeneity can act as a buffer and ensure at least some sub-population of cells is suited for a changing environment. A survival strategy employed, such as this, is known as “bet hedging”, especially given that LPS phase variants are likely maladapted for infection of a vertebrate hosts (De Jong et al., 2011; Grimbergen et al., 2015). Another possibility is that heterogeneity of BV and GV could provide an advantage during the transition from a vertebrate host back to the environment or vector reservoir where the selection against GVs may not be as strong.

In conclusion, we demonstrate the ability of several strains of *F. tularensis* to consistently form biofilm in a stochastic manner due to the emergence of GV strains. These results shed light on several important facets of *F. tularensis* biology and have implications for how this pathogenic bacterium may reside in the environment in a VBNC form. Current studies are underway to determine the genetic differences of our GV strains that were hyper biofilm producers. We hope that this will lead to a basis of biofilm formation and/or variance switching. Furthermore, we are examining the fitness and virulence potential of our *F. tularensis* strains that are hyper biofilm producers in both *in vitro* and *in vivo* assays. In addition to allowing us to understand the survival of *F. tularensis*, these studies on the role of biofilm and phase variation may lead to better medical countermeasures to prevent tularemia.

## Data Availability Statement

The original contributions presented in the study are included in the article/**Supplementary Material**. Further inquiries can be directed to the corresponding author.

## Author Contributions

KM and JB contributed conception and design of the study and wrote the manuscript. All authors participated in the experimentation, acquisition of data, analysis or interpretation of data for the work, read and approved the submitted manuscript.

## Funding

This research was performed while KM held a National Research Council Research Associate fellowship award supported by the Chem Bio Defense Research Associateship program sponsored by Defense Threat Reduction Agency. The research described herein was sponsored by the DTRA JSTO-CBD (project #CB10477 and CB10645) awarded to JB.

## Author Disclaimer

Opinions, interpretations, conclusions and recommendations are those of the authors and not necessarily endorsed by the U.S. Army.

## Conflict of Interest

The authors declare that the research was conducted in the absence of any commercial or financial relationships that could be construed as a potential conflict of interest.

## Publisher’s Note

All claims expressed in this article are solely those of the authors and do not necessarily represent those of their affiliated organizations, or those of the publisher, the editors and the reviewers. Any product that may be evaluated in this article, or claim that may be made by its manufacturer, is not guaranteed or endorsed by the publisher.

## Supplementary Material

Supplementary Figure S1 Culture density is not indicative of biofilm formation. **(A)** The OD_600_ values obtained after 7 d incubation (original, black) or 24 h (progeny, red) in CDM (growth) plotted against the OD_600_ value after crystal violet staining (biofilm). Samples were diluted to maintain the linear range of the measurement as necessary. Each dot represent a single well in an experiment. The least squares slope relating growth to biofilm is indicated by m. **(B)** Colony forming units were determined for samples that were either negative (black bars) or positive (grey bars) for biofilm formation by crystal violet staining at the time indicated. Error bars represent the standard error of the mean. N.D. indicates that no data were obtained at the time of sampling.

Supplementary Figure S2 Stochastic biofilm formation is observed at 25^o^C. **(A)** OD_600_ values for biofilm formation as assessed by crystal violet staining are displayed at the indicated time post inoculation. LVS was grown in CDM in a 96-well plate. **(B)** LVS was grown at 37^o^C in CDM then replica plated. Progeny were grown for 1 day at 25^o^C and biofilm formation was assessed using crystal violet stain. The red bar indicates the median value.

Supplementary Figure S3 Flattened cell morphology was observed in some instances. The biofilm of LVS biofilm was sampled at day 5. Image displayed is representative from multiple independent experiments.

Supplementary Figure S4 Fully virulent Type A and B isolate progeny maintain the ability to form biofilm upon sub-culture. Biofilm formation was assessed by crystal violet staining at 7 d post inoculation in FRAN 244 and FRAN255 (Type A and B isolate, respectively). Prior to staining, the original plates (top) were sub-cultured by replicate plating to create progeny plates (bottom). Progeny were assayed for biofilm development at 24 h post inoculation. Representative images are shown.

Supplementary Figure S5 OD_600_ Growth values of statically grown biofilm plates. The OD_600_ values obtained after over the course of 7 d static incubation in CDM are displayed. Black bar indicates the median value.

Supplementary Figure S6 Diverse colony morphologies are observed upon streaking cultures that formed biofilm. LVS and biofilm positive (Population 25) were streaked on chocolate agar and grown for 3 days at 37^o^C. Representative images are shown. Black arrow indicates large colony morphology, similar to wild-type while red arrows indicate the small colony morphology observed in grey variants.

Supplementary Figure S7 Diagram of the workflow used to identify biofilm positive isolates in this study. *F. tularensis* (LVS) was cultured in CDM for 7 d. Prior to staining, a glycerol stock was made from the contents of each well. **(A)** Wells identified as biofilm positive were assayed for biofilm formation at 3 d. Each bar represents the OD_600_ value corresponding to crystal violet straining of a biofilm positive population of bacteria obtained from independent experiments. Error bars represent SEM for 3 independent experiments. **(B)** Biofilm positive populations were streaked onto chocolate agar and incubated for 3 d to allow well defined colonies to appear. **(C)** Isolated colonies chosen at random from these populations were then inoculated in CDM and incubated again for 3 d. Prior to crystal violet staining, glycerol stocks were made to preserve the bacteria in each well. **(D)** Wells identified as biofilm positive were streak purified to obtain constitutive biofilm forming isolates. This process was used to obtain purified biofilm forming isolates in the LVS, FRAN244 and FRAN255 backgrounds.
