# Supplementary figures and images for: Phase Variation of LPS and Capsule Is Responsible for Stochastic Biofilm Formation in Francisella tularensis
Source: Front Cell Infect Microbiol. 2022 Jan 14;11:808550. doi: 10.3389/fcimb.2021.808550 (PMC8795689; doi:10.3389/fcimb.2021.808550)

**A**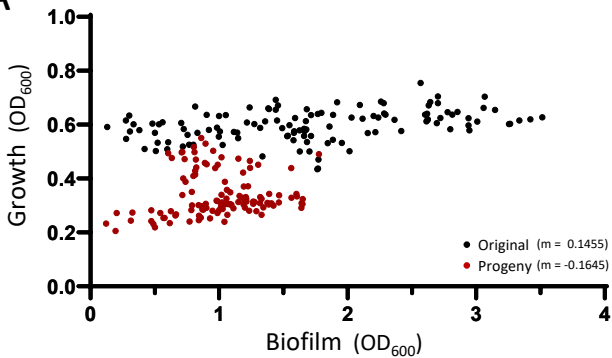**B**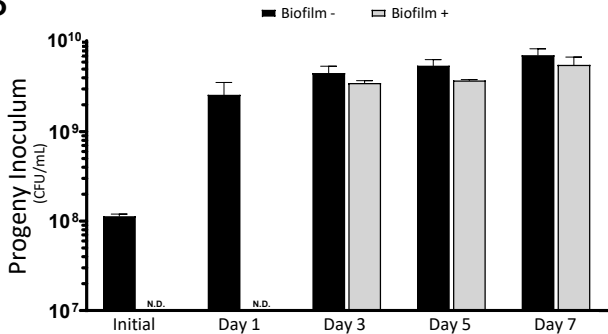

Supplement: Supplementary Figure S1 — Culture density is not indicative of biofilm formation. (A) The OD600 values obtained after 7 d incubation (original, black) or 24 h (progeny, red) in CDM (growth) plotted against the OD600 value after crystal violet staining (biofilm). Samples were diluted to maintain the linear range of the measurement as necessary. Each dot represent a single well in an experiment. The least squares slope relating growth to biofilm is indicated by m. (B) Colony forming units were determined for samples that were either negative (black bars) or positive (grey bars) for biofilm formation by crystal violet staining at the time indicated. Error bars represent the standard error of the mean. N.D. indicates that no data were obtained at the time of sampling. [file DataSheet_1.pdf]

**A**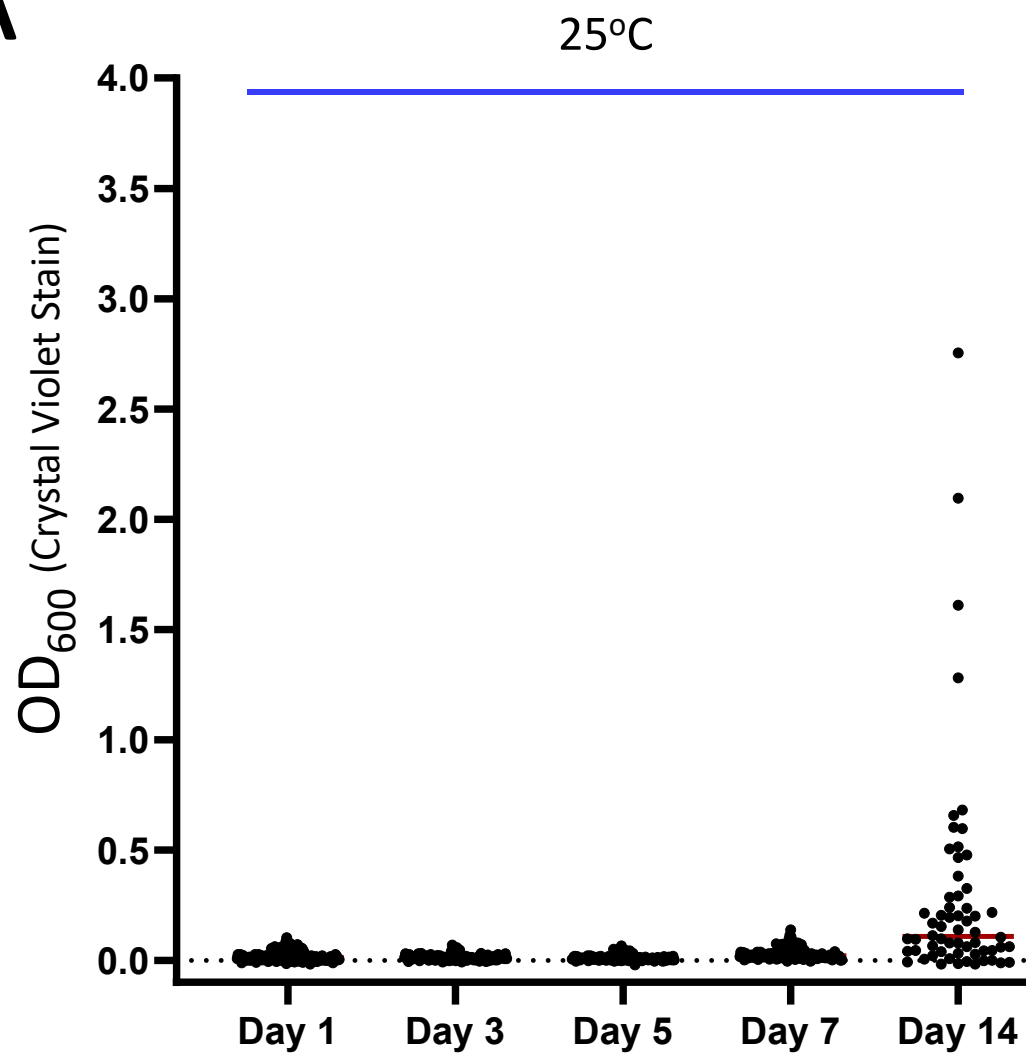**B**

37°C → 25°C

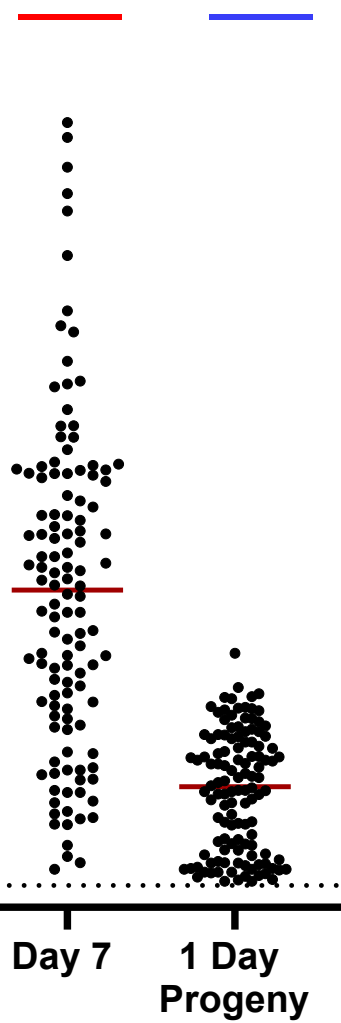

Supplement: Supplementary Figure S2 — Stochastic biofilm formation is observed at 25oC. (A) OD600 values for biofilm formation as assessed by crystal violet staining are displayed at the indicated time post inoculation. LVS was grown in CDM in a 96-well plate. (B) LVS was grown at 37oC in CDM then replica plated. Progeny were grown for 1 day at 25oC and biofilm formation was assessed using crystal violet stain. The red bar indicates the median value. [file DataSheet_2.pdf]

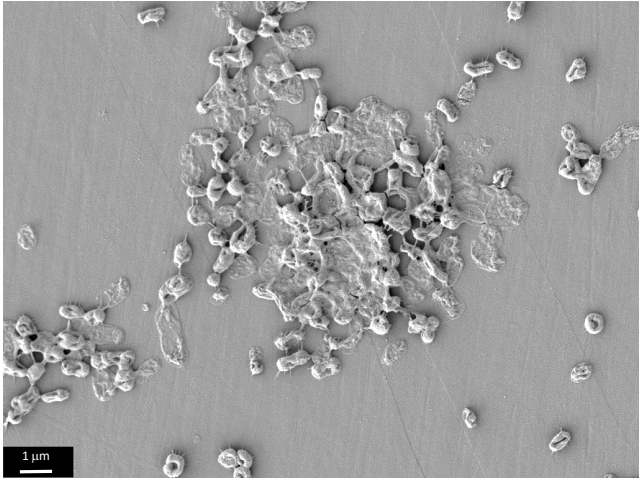

Supplement: Supplementary Figure S3 — Flattened cell morphology was observed in some instances. The biofilm of LVS biofilm was sampled at day 5. Image displayed is representative from multiple independent experiments. [file DataSheet_3.pdf]

Original  
day 7

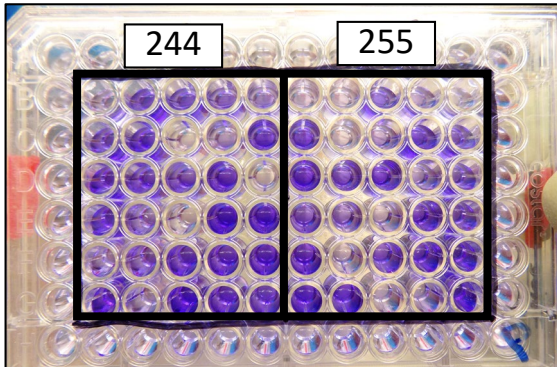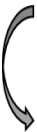

Progeny  
at 24 h

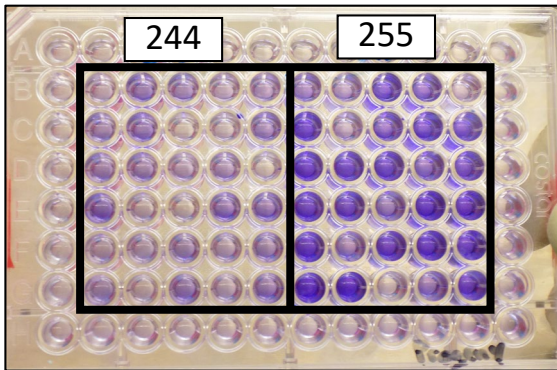

Supplement: Supplementary Figure S4 — Fully virulent Type A and B isolate progeny maintain the ability to form biofilm upon sub-culture. Biofilm formation was assessed by crystal violet staining at 7 d post inoculation in FRAN 244 and FRAN255 (Type A and B isolate, respectively). Prior to staining, the original plates (top) were sub-cultured by replicate plating to create progeny plates (bottom). Progeny were assayed for biofilm development at 24 h post inoculation. Representative images are shown. [file DataSheet_4.pdf]

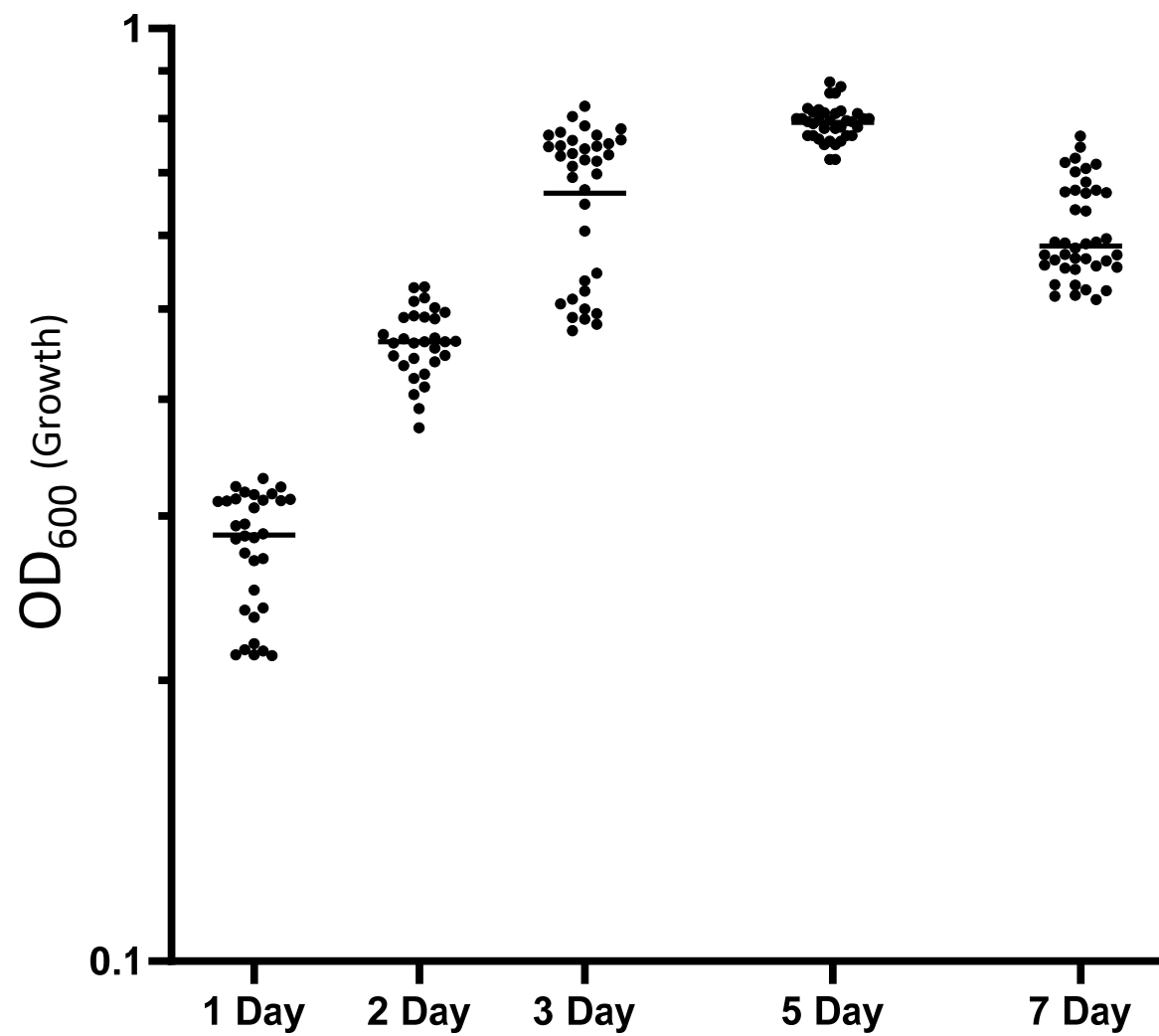

Supplement: Supplementary Figure S5 — OD600 Growth values of statically grown biofilm plates. The OD600 values obtained after over the course of 7 d static incubation in CDM are displayed. Black bar indicates the median value. [file DataSheet_5.pdf]

A

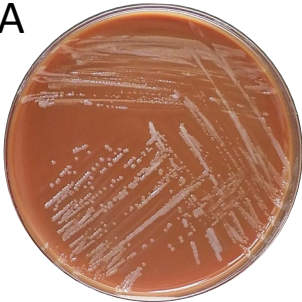

LVS wild-type

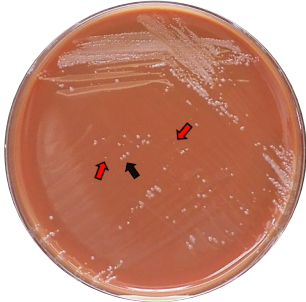

LVS Biofilm Positive  
Population 25

Supplement: Supplementary Figure S6 — Diverse colony morphologies are observed upon streaking cultures that formed biofilm. LVS and biofilm positive (Population 25) were streaked on chocolate agar and grown for 3 days at 37oC. Representative images are shown. Black arrow indicates large colony morphology, similar to wild-type while red arrows indicate the small colony morphology observed in grey variants. [file DataSheet_6.pdf]

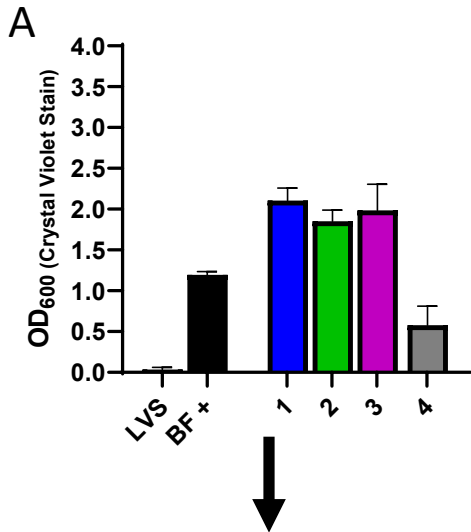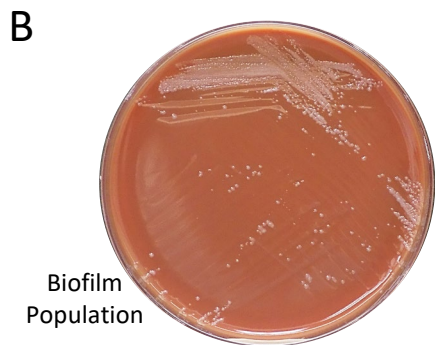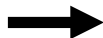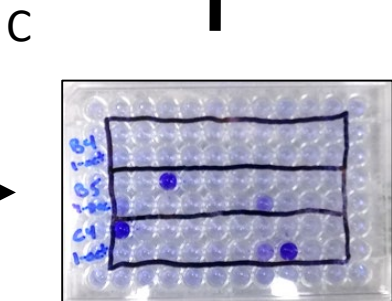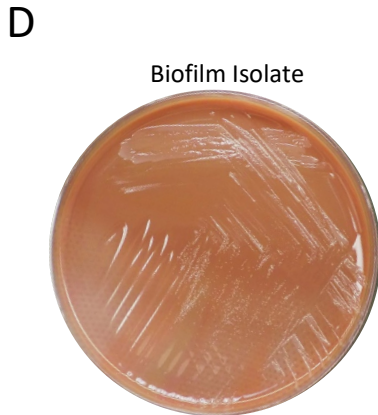

Supplement: Supplementary Figure S7 — Diagram of the workflow used to identify biofilm positive isolates in this study. F. tularensis (LVS) was cultured in CDM for 7 d. Prior to staining, a glycerol stock was made from the contents of each well. (A) Wells identified as biofilm positive were assayed for biofilm formation at 3 d. Each bar represents the OD600 value corresponding to crystal violet straining of a biofilm positive population of bacteria obtained from independent experiments. Error bars represent SEM for 3 independent experiments. (B) Biofilm positive populations were streaked onto chocolate agar and incubated for 3 d to allow well defined colonies to appear. (C) Isolated colonies chosen at random from these populations were then inoculated in CDM and incubated again for 3 d. Prior to crystal violet staining, glycerol stocks were made to preserve the bacteria in each well. (D) Wells identified as biofilm positive were streak purified to obtain constitutive biofilm forming isolates. This process was used to obtain purified biofilm forming isolates in the LVS, FRAN244 and FRAN255 backgrounds. [file DataSheet_7.pdf]
